# Acknowledgement to Reviewers of *Dentistry Journal* in 2019

**DOI:** 10.3390/dj8010013

**Published:** 2020-01-19

**Authors:** Dentistry Journal Editorial Office

**Affiliations:** MDPI, St. Alban-Anlage 66, 4052 Basel, Switzerland

The editorial team greatly appreciates the reviewers who have dedicated their considerable time and expertise to the journal’s rigorous editorial process over the past 12 months, regardless of whether the papers are finally published or not. In 2019, a total of 116 papers were published in the journal, with a median time to first decision of 23 days and a median time from submission to publication of 69 days. The editors would like to express their sincere gratitude to the following reviewers for their generous contribution in 2019:
Abdelaziz, MarwaKusnoto, BudiAbduo, JaafarKwon, Tae-YubAbrams, StephenLambert, MartijnAbubakr, Neamat HassanŁapińska, BarbaraAdekoya, Timothy O.Laranjo, MafaldaAfrashtehfar, KelvinLarsson, ChristelAfrashtehfar, Kelvin IanLauritano, DorinaAgrawal, PriyankaLee, Cheng-IAimetti, MarioLee, Sang JinAkinkugbe, Aderonke A.Lee, Sung-HoonAksel, HacerLerner, HenrietteAl-Horani, Rami A.Lezot, FredericAlmståhl, AnnicaLi, Jiao JiaoAl-Rudainy, DhelalLin, Hai-ShuAnastasiou, AntoniosLin, Yuh-YihAndrukhov, OlehLitschauer, BrigitteAngelova Volponi, AnaLiu, YuArany, PraveenLo Giudice, GiuseppeArdelean, Lavinia CosminaLo Muzio, LorenzoArendarczyk, NataliaLoewy, ZviArora, HimanshuLombardi, TommasoAsa’ad, FarahLombardo, GiorgioAspriello, Simone DomenicoLopez-Jornet, PiaAssari, ShervinLukomska-Szymanska, MonikaAvent, MinyonLunov, OlegAyoub, AshrafMa, SunyoungAyuso-Montero, RaúlMaddi, AbhiramBaghdadi, Ziad D.Magán-Fernández, AntonioBahari-Chopiuk, NasrinMagiorkinis, EmmanouilBaltriukiene, DaivaMaiorana, CarloBanas, JeffreyMakvandi, PooyanBarbeck, MikeManton, DavidBarberis, FabrizioMarcal, HelderBarnett, TonyMarcé-Nogué, JordiBatchelor, PaulMarshman, ZoeBekes, KatrinMartins, FranciscoBel’skaya, LyudmilaMarto, Carlos MiguelBernardello, FabioMascitti, MarcoBernardi, SaraMatys, JacekBettini, GiordanaMaurin, Jean-ChristopheBian, LiMayberry, MelanieBorzabadi-Farahani, AliMays, Jacqueline W.Bourgeois, DenisMeister, JörgBrantley, WilliamMendes, JoaquimBright, RichardMerigo, ElisabettaBritt, EileenMertas, AnnaBrowar, Andrew W.Meurman, JukkaBuckley, M.Mezzomo Collares, FabrícioBullon, PedroMicutz, MarinCalvo-Guirado, José LuisMihelogiannakis, DimitriosCampus, GuglielmoMitsea, AnastasiaCarey, CliftonMobilio, NicolaCarrilho, EuniceMoore, RodCarrouel, FlorenceMorra, MarcoCasimiro, Maria HelenaMoussa, DinaCerra, FrankNalliah, RomeshCervino, GabrieleNicholi Vorsa, Nicholi VorsaChacko, Jenu VargheseNicolescu, Mihnea-IoanChang, MeichiNieminen, PenttiChen, JunningOgi, KazuhiroChen, Min-HueyOgórek, RafałCheung, Gary Shun PanOwens, JanineChi, GeraldPadial-Molina, MiguelChladek, GrzegorzPagano, StefanoChoi, Sung-HwanPalma, Paulo JChrysanthakopoulos, Nikolaos A.Palmer, Robert J.Chu, Chun HungPalomo, LeenaCicciù, MarcoPang, Eun-KyoungColeman, NicholaPapadopoulos, MoschosColetta, RicardoPapadopoulou, AlexandraContaldo, MariaPar, MatejCorbella, StefanoPark, Chan HoCui, ZhibinPark, Ji-ManCvikl, BarbaraPark, Jun-BeomCvikl, Doz. BarbaraPatel, UpenCzarnecka, BeataPathak, AshishDalessandri, DomenicoPatini, RomeoDammaschke, TillPeroš, KristinaDanesh-Sani, Seyed AmirPilo, RaphaelDarvell, BrianPopat, HashmatDe Biase, AlbertoPorcheri, CristinaDelimont, NicolePorojan, LilianaDellavia, ClaudiaPorritt, JennyDerman, Sonja H. M.Pramudita, Jonas A.Di Blasio, AlbertoQasim, SaadDi Carlo, GabrieleQuinn, BarryDi Stasio, DarioRamaswamy, RavishankarDiogo, PatríciaRandall, CameronDioguardi, MarioRead-Fuller, AndrewDiomede, FrancescaReddy, SakamuriDionysopoulos, DimitriosRener-Sitar, KsenijaDoerrler, WilliamReukov, VladimirDonchin, MilkaReychler, HervéDziedzic, ArkadiuszRibeiro, ApoenaEhrlich, YgalRíos-Santos, Jose VicenteEick, SigrunRitz, UlrikeEkuni, DaisukeRobertson, AgnetaEl-Bialy, TarekRodríguez-Lozano, Francisco JavierEl-Housseiny, AzzaRoehling, Stefan K.Eliades, GeorgeRogers, JohnEliades, TheodoreRoggendorf, Matthias JEmmanouil, DimitrisRomano, FedericaEnrico, Gherlone FeliceRongo, RobertoFakhouri, Walid D.Rudolf, RebekaFarronato, DavideRudolph, HeikeFelix Gomez, Grace GomezRusu, Laura CristinaFernandes, Maria GoretiRuys, AndrewFernández-Revelles, Andrés B.Ryabov, IgorFerrarotti, FrancescoSaadi, IrfanFerreira, Manuel MarquesSabbagh, Heba JafarFigueiral, Maria HelenaSafavi, KamranFiner, YoavSahrmann, PhilippFiorillo, LucaSato, TakuichiFlaherty, KevinSchiavino, DomenicoFornaini, CarloScoffield, JessicaForner, LeopoldoScribante, AndreaFoster Page, Lyndie A.Sefat, FarshidFox, SimonSegura, Juan-JoseFrança, RodrigoSekiguchi, YuFu-Mei, HuangSeok, HyunFusco, VittorioSerpico, RosarioGalal, SuzanneShah, Parag KGalli, SilviaShahmoradi, MahdiGalluccio, GabriellaShanmugam, MuruganandanGao, XinShort, LeonieGarner, AngieSingha, SubhankarGhezzi, Elisa M.Singhrao, Sim K.Gibson, FrankSips, PatrickGlavina, DomagojSivasubramanian, KathyayiniGlazar, IrenaSkopouli, FotiniGlockner, KarlSones, Amerian D.Gorseta, KristinaSpagnuolo, GianricoGoto, TakaharuSrinivasan, SowmyaGregory, Richard LStaderini, EdoardoGrimm, Wolf-DieterSUKUMARAN, ANILGrzech-Leśniak, KingaSung, Wen-WeiGudey, Shyam KumarSurdacka, AnnaGutierrez-Perez, Jose-LuisSurdacki, AndrzejHack, GarySuzuki, Jon B.Han, PingpingSwain, MichaelHanisch, MarcelSwann, Brian JeffreyHaraszthy, VioletSzczepanski, CarolineHaynes, DavidTallarico, MarcoHemphill, Jean CroceTestori, TizianoHerten, MonikaThikkurissy, SaratHeyman, Richard EThorsen, AndreasHirata, RonaldoTimothe, PeggyHoover, JayTomaszewska, EwaHorowitz, Robert A.Torres-Lagares, DanielHowe, BrianTroedhan, AngeloHristov, IlianTseng, Yu-ChuanHughes, Christopher V.Tsuchida, SachioHülsmann, MichaelTu, Ming-GeneIngendoh-Tsakmakidis, AlexandraTuna, TaskinInnes, NicolaUpadhyaya, JasbirIrwin, JenniferVandenberghe, BartIsola, GaetanoVassiliou, Leandros-VassiliosJanakiraman, HarinarayananVaz, Maria De Fátima ReisJeng, Jiiang-HueiVaz, PaulaJinno, YoheiVeerkamp, JaapJohnson, MareeVerket, AndersJordana, FabienneVettore, MarioJoseph, SusanVissink, ArjanJu, XiangqunVozza, IoleKaisarly, DaliaWalsh, Laurence JKameyama, AtsushiWalter, ChristianKaminski, RafalWang, HaoyuKanamoto, TaiseiWang, Jeff CW.Kanno, TakahiroWang, KaiKanzaki, HiroyukiWashio, AyakoKanzow, PhilippWhite, Raymond P.Karpiński, Tomasz MWieckiewicz, MieszkoKatic, VisnjaWierichs, RichardKebschull, MoritzWilson, MichaelKhurshid, ZohaibWunsch, Patrice B.Kim, Hae-YoungXiao, LiKim, J.-W.Xie, QianKim, Jin-BomYamagata, KenjiKim, Seong-GonYamaguchi, SatoshiKim, Young-jaeYang, ChengwuKlontzas, MichaelYeh, Chih-KoKniha, KristianYeung, Andy Wai KanKontonasaki, EleanaYuan, KuoKorsunsky, Alexander M.Yuan, SiyangKosuke, NozakiYumoto, HiromichiKranz, StefanZafar, Muhammad SohailKrystofiak, TomaszZhan, LingKujan, OmarZiebart, ThomasKumar, PRAKASH


